# Neurological manifestations of polyarteritis nodosa: a tour of the neuroaxis by case series

**DOI:** 10.1186/s12883-021-02228-2

**Published:** 2021-05-21

**Authors:** Cathra Halabi, Erika K. Williams, Ramin A. Morshed, Mauro Caffarelli, Christine Anastasiou, Tarik Tihan, Daniel Cooke, Adib A. Abla, Christopher F. Dowd, Vinil Shah, Sharon Chung, Megan B. Richie

**Affiliations:** 1 Department of Neurology, Neurovascular Division, University of California, 505 Parnassus Avenue, Box 0114, San Francisco, California 94143 USA; 2 Weill Institute for Neurosciences, University of California, San Francisco, California USA; 3 Department of Neurology, Partners Healthcare, Boston, MA USA; 4 Department of Neurosurgery, University of California, San Francisco, California USA; 5 Department of Neurology, University of California, San Francisco, California USA; 6 Russell/Engleman Rheumatology Research Center, University of California, San Francisco, California USA; 7 Department of Pathology, Neuropathology Division, University of California, San Francisco, California USA; 8 Department of Neurointerventional Radiology, University of California, San Francisco, California USA; 9 Department of Neuroradiology, University of California, San Francisco, California USA; 10 Department of Neurology, Neurohospitalist Division, University of California, San Francisco, California USA

**Keywords:** Polyarteritis nodosa, Spinal artery aneurysm, Intracranial aneurysm, Multidisciplinary, Case series

## Abstract

**Background:**

Heterogenous central nervous system (CNS) neurologic manifestations of polyarteritis nodosa (PAN) are underrecognized. We review three cases of patients with PAN that illustrate a range of nervous system pathology, including the classical mononeuritis multiplex as well as uncommon brain and spinal cord vascular manifestations.

**Case presentation:**

Case 1 presented with mononeuritis multiplex and characteristic skin findings. Case 2 presented with thunderclap headache and myelopathy due to spinal artery aneurysm rupture. Both patients experienced disease remission upon treatment. Case 3 presented with headache and bulbar symptoms due to partially thrombosed intracranial aneurysms, followed by systemic manifestations related to visceral aneurysms. She demonstrated clinical improvement with treatment, was lost to follow-up, then clinically deteriorated and entered hospice care.

**Conclusions:**

Although the peripheral manifestations of PAN are well-known, PAN association with CNS neurovascular disease is relatively underappreciated. Clinician awareness of the spectrum of neurologic disease is required to reduce diagnostic delay and promote prompt diagnosis and treatment with immunosuppressants.

## Background

Polyarteritis nodosa (PAN) is a primarily medium-caliber artery vasculitis that leads to diverse clinical manifestations depending on the affected vascular territories. Arterial wall injury can cause aneurysms, vessel irregularity, hemorrhage, or thrombosis with downstream ischemia, and pathology demonstrates segmental transmural inflammation with associated fibrinoid necrosis [1]. The vasa nervorum, the peripheral nervous system (PNS) arterial supply, is one of the most frequently involved sites and up to 85% of PAN patients develop peripheral neuropathy [2]. Classically, patients develop mononeuritis multiplex, though symmetric distal polyneuropathy, radiculopathy, plexopathy, and cranial neuropathy can also occur [2, 3].

PAN can also affect the central nervous system (CNS) and is recently reported to occur in 4.6% of patients [2–4]. Older studies report CNS involvement as an advanced finding in 20–45% of presumptive PAN cases [3, 5]. Case reports describe PAN-associated spinal artery aneurysms and related complications such as hemorrhage and cord compression [6, 7]. Raising awareness of disease heterogeneity is critical because prompt immunosuppressive therapy improves outcomes [1]. To highlight the spectrum of nervous system presentations we review 3 cases of PAN impacting the vasa nervorum, spinal arteries, and intracranial arteries.

## Case presentation

### Case 1

A 59-year-old man with minimal past medical history developed subacute bilateral foot drop followed by diffuse paresthesias, fingertip discoloration, and proximal spread of limb pain and weakness associated with fatigue and 9 kg weight loss. He initially presented with bilateral lower extremity edema, then developed associated severe pain limiting ambulation. Two weeks later, he noticed left hand numbness and allodynia. Within days, his left fingers appeared cyanotic and he began dropping items due to weakness. He was advised by a local provider to wear a rigid cast over his lower extremities, and after cast removal 1 week later the patient had new bilateral foot drop. He had also developed numbness and weakness in the right hand. Six weeks after symptom onset, he presented to a tertiary care center for further evaluation.

General exam revealed fingerpad necrosis, bullae, and extremity retiform purpura and ulcerations. Neurologic exam demonstrated asymmetric hand weakness, bilateral gastrocnemius and extensor hallucis longus plegia, and patchy sensation loss. Specifically, in the upper extremities he had mild (graded 4) right triceps weakness, mild left wrist extensor weakness, mild finger extensor and flexor weakness bilaterally, mild right intrinsic hand muscle weakness and moderate (graded 3) left intrinsic hand muscle weakness. In the lower extremities, he could not dorsiflex nor plantarflex the feet or toes. Laboratory studies showed mild peripheral leukocytosis and anemia, mild transaminitis, erythrocyte sedimentation rate (ESR) 85 mm/hr., and C-reactive protein (CRP) 121 mg/L. Unremarkable labs included renal function, urinalysis, human immunodeficiency virus (HIV) and hepatitis C virus testing, cryoglobulins, and antinuclear (ANA) and antineutrophil cytoplasmic antibodies (ANCA). Hepatitis B serologies demonstrated prior exposure with undetectable viral load.

Nerve conduction studies (NCS) and electromyography (EMG) were performed. In the upper extremities, bilateral median sensory nerve action potentials (SNAPs) were absent, the bilateral ulnar and radial SNAPs demonstrated normal peak latencies with reduced amplitudes, and bilateral median compound motor action potentials (CMAPs) demonstrated reduced amplitudes with slowed conduction velocities (CV). In the lower extremities, left sural and superficial peroneal SNAPs were absent, and the left peroneal and tibial CMAPs were absent. Concentric needle EMG of select muscles of the left upper extremity demonstrated fibrillation potentials in the triceps, flexor carpi ulnaris, extensor digitorum communis, first dorsal interosseus (FDI), and abductor pollicis brevis (APB). On activation, the left triceps and FDI demonstrated normal motor unit action potential (MUP) morphology with reduced recruitment patterns; no motor units could be recruited in the APB. Concentric needle EMG of select muscles in the left lower extremity demonstrated fibrillation potentials in the left tibialis anterior, medial head of the gastrocnemius, and short head of the biceps femoris. On activation, no motor units could be recruited in the tibialis anterior or the medial head of the gastrocnemius. Findings from the clinical presentation including EMG/NCS were consistent with a severe, acute, axonal, multifocal sensorimotor polyneuropathy (mononeuritis multiplex). Sural nerve and gastrocnemius muscle biopsies revealed vasculitis of small to medium-sized arteries. The patient was diagnosed with PAN and treated with oral cyclophosphamide (150 mg/day, body weight 66.9 kg) and prolonged prednisone taper for remission induction (60 mg daily tapered over months). Cyclophosphamide was switched to mycophenolate mofetil (MMF) 1500 mg twice daily for maintenance. He had a relapse upon taper of MMF to 500 mg twice daily and was retreated with high dose steroids, cyclophosphamide (100 mg/day for 3 months), then MMF 1000 mg twice daily. For the past decade, he has been maintained on MMF only and continues lamivudine 150 mg daily to prevent hepatitis B reactivation. He leads an active, independent life despite residual asymmetric foot weakness.

### Case 2

**Fig. 1:**
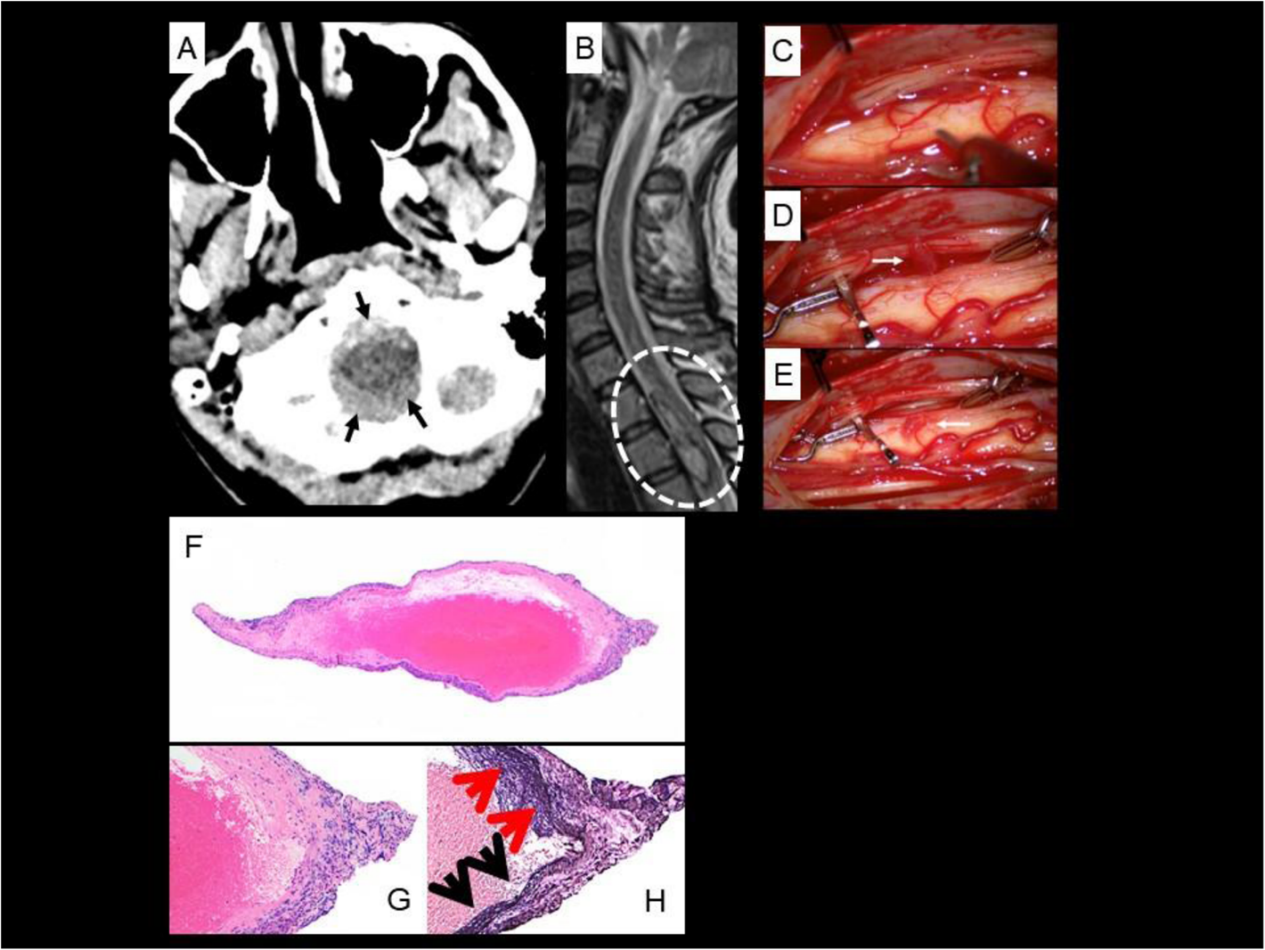
Case 2. **a** Axial non-contrast CT image at the level of the foramen magnum shows diffuse subarachnoid hemorrhage (solid arrows). **b** Sagittal T2W MRI image of the spine shows intradural hemorrhage in the upper thoracic spine (dashed oval) compressing and posteriorly displacing the adjacent thoracic cord. **c-e** Intraoperative images of fusiform thoracic spinal artery aneurysm resection. **d** A large fusiform aneurysm was identified (arrow) and isolated with 2 clips placed on either side along the parent vessel. **e** The fusiform aneurysm and small segment of posterior spinal artery was resected (arrow) using microsurgical technique and separated from the arachnoid of the cord. **f** Low power magnification view of the biopsy from the posterior spinal artery branch showing disruption of the vessel wall (original X40). **g** High power view of the vessel wall with lymphocytic infiltration and destruction of vessel wall, consistent with vasculitis (original × 100). **h** Elastic Van-Gieson stain demonstrating presence of elastic lamina in the lower half of the vessel wall (black arrows) and its destruction on the upper half of the vessel wall (red arrows, original × 100)

**Fig. 2:**
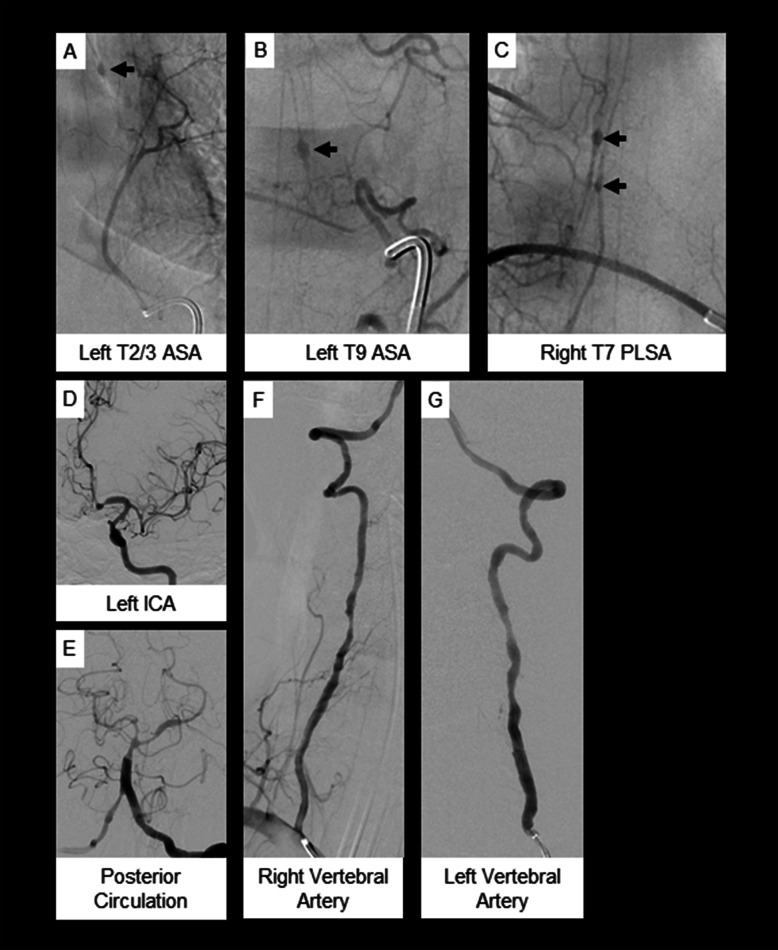
Case 2. **a-c** Spinal angiogram and (**d-g**) cerebral angiogram. **a** & **b** Multiple fusiform aneurysms (solid arrows) arising from the anterior spinal artery and (**c**) posterior lateral spinal artery. **d-g** Findings consistent with diffuse intra- and extracranial vasculopathy with multifocal narrowing and irregularity

Seven days after delivery of a term infant, a 35-year-old woman with history of gestational diabetes and multinodular goiter presented with thunderclap headache followed by acute lower extremity paralysis. She delivered a healthy infant via uncomplicated Cesarean-section and was discharged to home. Six days after delivery, she developed thunderclap occipital headache with chest pain and presented to the local emergency department. Her presenting vital signs were normal with a blood pressure of 126/85. A brain MRI without gadolinium was unremarkable. The following day, as she was undergoing diagnostic CT and CT angiography (CTA) of the head and neck, she developed severe mid-back pain followed quickly by bilateral lower extremity weakness and numbness on the order of minutes. The related head CT images then revealed acute subarachnoid hemorrhage in the right sylvian fissure, basal cisterns and in the visualized portion of the anterior aspect of the cervical spine (Fig. 1) and head and neck CT angiography was interpreted as normal. Emergent spine magnetic resonance imaging (MRI) revealed an intradural, extramedullary thoracic spine hematoma with cord compression (Fig. 1) which was surgically decompressed. Digital subtraction angiography (DSA) revealed diffuse intracranial and extracranial vasculopathy (Fig. 2). The patient was diagnosed with reversible cerebral vasoconstriction syndrome and transferred to a tertiary care center.

Unremarkable studies included INR, prothrombin time, activated partial thromboplastin time, ESR, CRP, ANCA, anti-Smith and ribonucleic protein antibodies, double-stranded deoxyribonucleic acid antibodies, cryoglobulins, hepatitis serologies, HIV, syphilis, beta-d-glucan, and galactomannan testing. ANA titer was 1:640 with mixed diffuse and speckled pattern. Repeat MRI of the spine 6 days after the first one revealed expected changes following surgical hematoma evacuation but also new spinal cord hemorrhages at multiple levels. Meticulous brain and total spine DSA 2 days later demonstrated intracranial and extracranial vasculopathy and small fusiform aneurysms of the anterior spinal and posterolateral spinal arteries at multiple thoracic levels (Fig. 2) which were not amenable to endovascular treatment. Abdominal angiogram demonstrated small aneurysms arising from the common hepatic artery and bilateral renal arteries.

A T6–8 laminectomy was performed with clipping and resection of the right T7 posterolateral aneurysm (Fig. 1). Tissue evaluation revealed vessel wall fibrinoid necrosis with inflammatory cell infiltration prompting diagnosis of PAN (Fig. 1). She received 5 days of IV solumedrol followed by oral prednisone taper and 6 months of 15 mg/kg IV cyclophosphamide (54.7 kg body weight), then remission maintenance with azathioprine 2 mg/kg and low dose oral prednisone. At the time of discharge from her hospitalization, she had normal mental status, cranial nerve, and upper extremity sensorimotor examinations; lower extremity strength was graded as 0 or 1 on the right and 4 on the left. Seven months later she had regained distal dorsiflexion and plantar flexion (2 and 4, respectively) on the right and improved strength in most muscle groups to 5 on the left with the exception of knee extension which remained 1. She had improved but persisting sensory changes and intact bowel and bladder function. Review of records indicate further modest improvement in strength 2 years following the index injury. Her American Spinal Injury Association (ASIA) scale score is extrapolated to be ASIA D (motor function preserved below injury level with at least half of key muscles below injury level with strength graded at least 3).

### Case 3

**Fig. 3:**
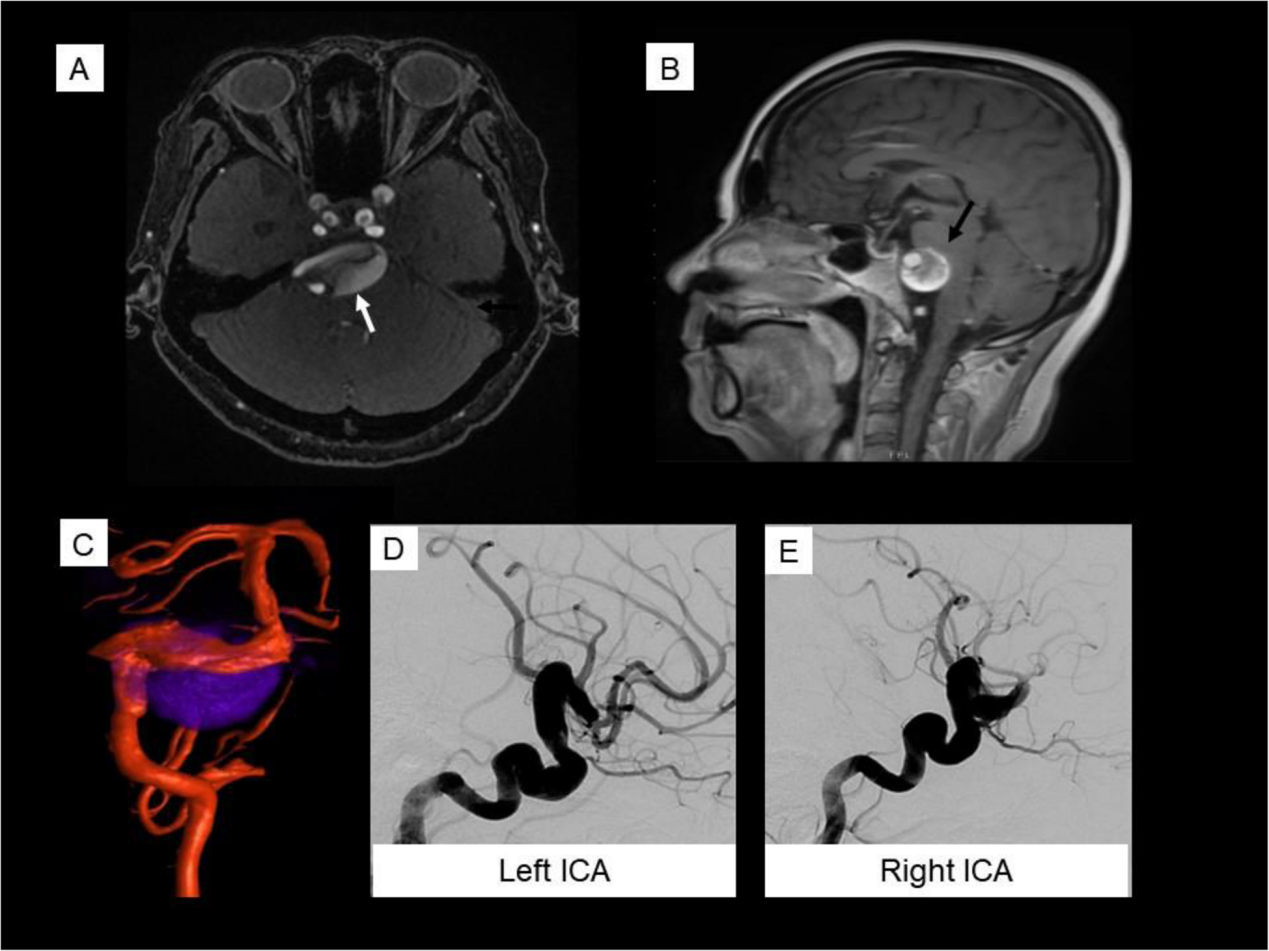
Case 3. Giant vertebrobasilar aneurysm. Gadolinium-enhanced MR angiogram (**a**) and Sagittal T1 sequence (**b**) demonstrate a partially thrombosed vertebrobasilar aneurysm (white arrow in A). Note the significant mass effect on the pons (black arrow). Fused conventional angiography 3D reconstruction and MR of the vertebrobasilar system with dolichoectatic vessels (red) and outline of partially thrombosed aneurysm (blue) (**c**). Conventional angiography with left (**d**) and right (**e**) lateral carotid injections demonstrating arteriomegaly of the anterior circulation

**Fig. 4:**
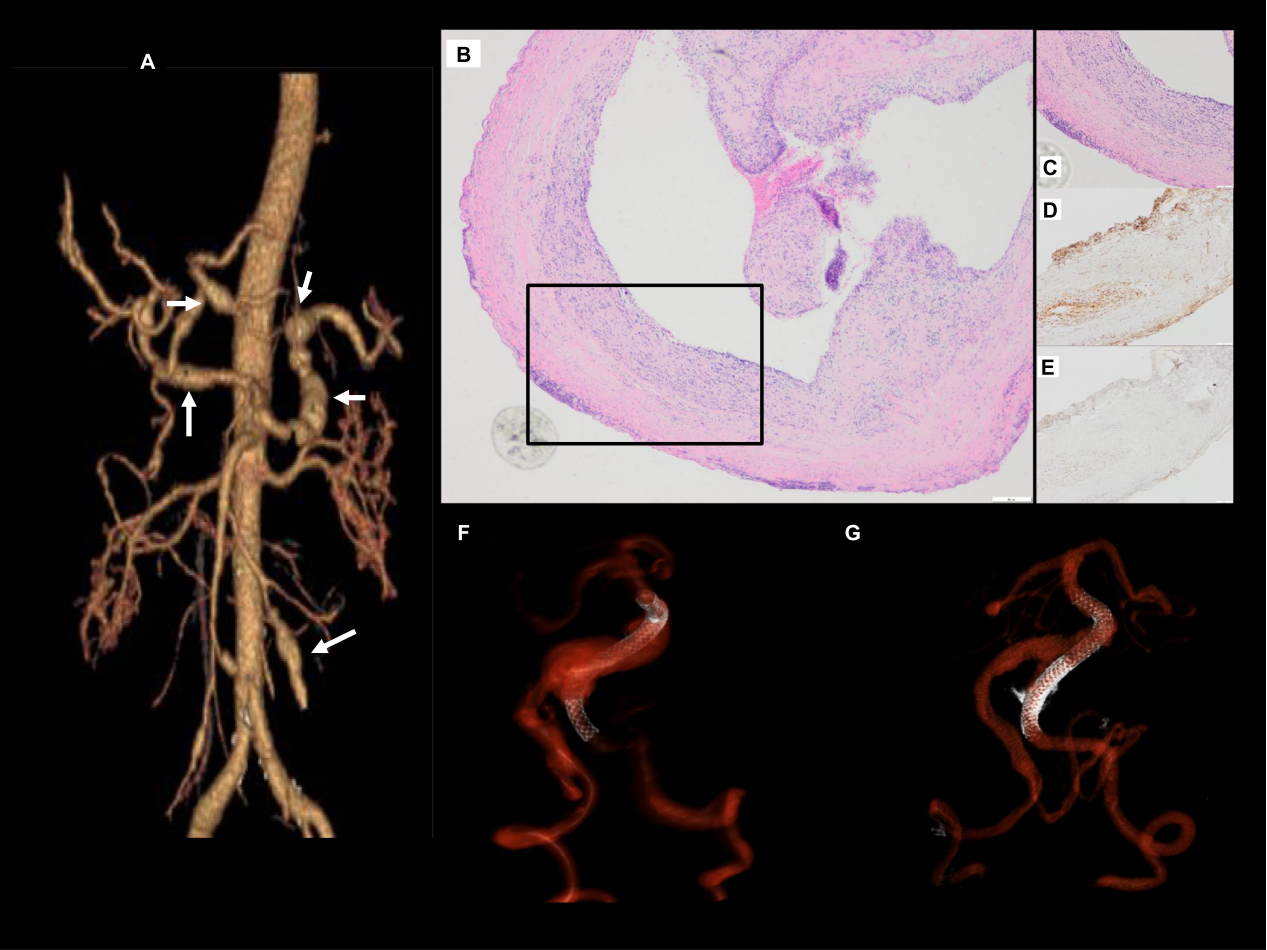
Case 3. **a** CT angiography 3D reconstruction demonstrating multiple fusiform aneurysms of the hepatic, splenic, and superior mesenteric arteries (arrows). **b** H&E staining shows the cross-section of the aneurysm tip (original × 40). **c** Medium power microscopic images showing inflammation involving the aneurysm wall (original × 100). Immunohistochemical staining with the antibodies against p65 subunit of NFkB (**d**, original × 100) and TNFa (**e**, original × 100**)** shows focal and strong positive staining within the inflammatory infiltrates as expected. **f, g** 3D reconstruction of vertebrobasilar system after conventional angiography and PED deployment into the left vertebral artery (**f**), and repeat procedure 3 months later with second PED demonstrating overlapping stents and smaller aneurysm (**g**). Note reduction of flowing portion of the aneurysm sac as contemporaneously performed MR demonstrated continued enlargement of the thrombosed aneurysm sac with mass effect.

A 56-year-old woman with diabetes and hypertension developed 3 months of progressive headache, diplopia and dysphagia. Brain MRI and MR angiogram revealed diffuse arteriopathy, a left posterior inferior cerebellar artery (PICA) aneurysm, and partially thrombosed giant vertebrobasilar aneurysm with mass effect on the brainstem (Fig. 3). The patient underwent left occipital artery to anterior inferior cerebellar artery bypass and left PICA aneurysm clipping and excision. She then developed abdominal pain and imaging revealed visceral aneurysms (Fig. 4) and progressive thrombotic disease with visceral infarcts. Hepatitis serologies, HIV, treponemal antibody, and QuantiFERON-TB Gold studies were negative; serum ANCA were not tested. CRP was 10.9 mg/L and ESR 60 mm/hr. Retrospective review of an occipital artery specimen revealed adventitial and intimal inflammation and focal disruption of the elastic lamina (Fig. 4).

She was diagnosed with PAN and started on high dose steroids with gradual improvement. She underwent two vertebrobasilar junction Pipeline embolization device (PED) [8] deployments at months 1 and 5 with interim endovascular coil occlusion of the right intradural vertebral artery at PICA origin due to symptomatic growing vertebrobasilar aneurysm (Fig. 4). She was non-adherent to planned cyclophosphamide infusions and received a single dose of 1000 mg (81.1 kg body weight) 5 months after surgery. Cyclophosphamide was converted to an oral regimen to encourage adherence but she did not take this medication. After a period of clinical deterioration, the patient entered hospice care.

## Discussion and conclusions

PAN is a necrotizing vasculitis of primarily medium-sized arteries and is categorized as having systemic, cutaneous, or secondary forms. Secondary PAN can develop in the setting of hepatitis B or C infection or hairy cell leukemia [9–11]. The prevalence of PAN is approximately 30 per million and may be declining due to hepatitis B vaccination [1]. Characteristics of systemic PAN that differentiate it from other vasculitides include its sparing of the lungs, restriction to the arterial circulation, negative ANCA, and lack of granulomatous inflammation [12]. Mononeuritis multiplex is the most common neurologic manifestation, affecting up to 70% of patients [2]. Other described PNS syndromes include plexopathy, radiculopathy, cranial neuropathy, and symmetric distal polyneuropathy [13]. Tissue specimen provides definitive diagnosis. Combined nerve and muscle biopsy is recommended to increase diagnostic yield. Preoperative NCS, EMG, and peripheral nerve MRI can confirm axonal neuropathy and identify biopsy targets with the highest diagnostic yield. The differential diagnosis for PAN includes infection (e.g., HBV, HCV, HIV, tuberculosis, and endocarditis), thromboembolic disorders (e.g., anti-phospholipid antibody syndrome), and structural vascular disorders (e.g., fibromuscular dysplasia, segmental arterial mediolysis). Classification criteria for polyarteritis nodosa were presented by the American College of Rheumatology in 1990 but were intended to facilitate research, not diagnosis [14, 15].

CNS manifestations of PAN are rare and present diagnostic challenges [16]. Cases 2 and 3 illustrate the potential for diagnostic delay due to low initial suspicion for vasculitis. Most patients with active PAN have elevated inflammatory markers, thus ESR and CRP are helpful screening tests in patients presenting with CNS arteriopathy secondary to PAN. Normal inflammatory markers should not exclude consideration of vasculitis in the appropriate clinical context (Case 2). Obtaining tissue diagnosis can be challenging in CNS PAN due to potential morbidity of biopsy targets. Case 2 demonstrates the posterolateral spinal artery may be an accessible target in cases with cord involvement, while Case 3 exemplifies the importance of tissue review in fulminant presentations of brain aneurysms.

Neurovascular consequences of PAN are frequently life threatening but can be mitigated with appropriate treatment. Idiopathic generalized PAN is treated with systemic immunosuppression such as glucocorticoids and cyclophosphamide, followed by less toxic medications such as methotrexate or azathioprine. Neurologic PAN is considered organ-threatening and typically warrants cyclophosphamide. Aneurysms can pose unique management challenges due to friable inflammatory vessel wall changes. Successful flow-diverting PED placement has been reported and was a strategy used in Case 3 [8]. Cutaneous PAN is managed with topical therapies with consideration of systemic immunosuppression depending on disease severity. Finally, HBV-associated PAN is best treated with antiretrovirals.

This case series demonstrates the spectrum of neurologic PAN. Clinicians should consider systemic vasculitis in the evaluation of any fulminant neurologic presentation, and tissue diagnosis is essential. Normal inflammatory markers should not delay seeking biopsy targets in the appropriate clinical context. Once PAN is diagnosed, aggressive immunosuppressive treatment is warranted to prevent its significant morbidity and mortality.

## Data Availability

The authors declare that all data supporting the findings of this study are available within the article.

## Abbreviations

ANA: Antinuclear antibodies
ANCA: Antineutrophil cytoplasmic antibodies
APB: Abductor pollicis brevis
CMAP: Compound muscle action potential
CNS: Central nervous system
CRP: C-reactive protein
CT: Computed tomography
CV: Conduction velocity
DSA: Digital subtraction angiography
EDC: Extensor digitorum communis
EMG: Electromyography
FCU: Flexor carpi ulnaris
FDI: First dorsal interosseus
H&E: Hematoxylin and eosin
HBV: Hepatitis B virus
HCV: Hepatitis C virus
HIV: Human immunodeficiency virus
IHC: Immunohistochemistry
IV: Intravenous
Kg: Kilogram
Mg: Milligram
MMP: Mycophenolate mofetil
MR: Magnetic resonance
MRI: Magnetic resonance imaging
MUP: Motor unit action potential
NCS: Nerve conduction studies
NFkB: Nuclear factor kappa B
PAN: Polyarteritis nodosa
PED: Pipeline embolization device
PICA: Posterior inferior cerebellar artery
PNS: Peripheral nervous system
SNAP: Sensory nerve action potential
T2W: T2 weighted
TA: Tibialis anterior
TNFa: Tumor necrosis factor alpha
